# Draft Genome Sequences of Two Multidrug-Resistant Sequence Type 405 Escherichia coli Isolates without Clinical Infection

**DOI:** 10.1128/MRA.00508-21

**Published:** 2021-08-05

**Authors:** Amanda Chamieh, Rita Zgheib, Sabah El-Sawalhi, Eid Azar, Jean-Marc Rolain

**Affiliations:** a Division of Infectious Diseases, Saint George Hospital University Medical Center, Beirut, Lebanon; b Aix-Marseille Université, Institut de Recherche pour le Développement, UMR Microbes Evolution Phylogeny and Infections, Marseille, France; c Institut Hospitalo-Universitaire Méditerranée Infection, Marseille, France; d Aix-Marseille Université, Institut de Recherche pour le Développement, Service de Santé des Armées, AP-HM, UMR Vecteurs Infections Tropicales et Méditerranéennes, Marseille, France; University of Maryland School of Medicine

## Abstract

We present the genome sequences of two carbapenemase-producing sequence type 405 Escherichia coli clinical isolates, strains Marseille-Q1950 and Marseille-Q1951. The isolates were obtained 1 month apart during the patient’s hospitalization in Lebanon, in May (Marseille-Q1950) and June (Marseille-Q1951) 2019. The genome sizes of strains Marseille-Q1950 and Marseille-Q1951 were 5,181,515 bp and 5,213,451 bp, respectively.

## ANNOUNCEMENT

We isolated two multidrug-resistant Escherichia coli strains, Marseille-Q1950 and Marseille-Q1951, from the same patient in May and June 2019, respectively, during his stay in the intensive care unit (ICU) at Saint George Hospital University Medical Center (SGHUMC) (Beirut, Lebanon). In May 2019, we obtained the first isolate when the patient’s central femoral catheter was removed and sent for culture. In June 2019, we obtained the second isolate with a routine infection control surveillance and screening protocol in the SGHUMC ICU. The SGHUMC infection prevention and control division has a constitutional institutional review board waiver to retrieve relevant records for patients with pathogens of high importance.

The two samples were cultured on MacConkey agar for 24 h, incubated at 37°C, and then identified as E. coli by matrix-assisted laser desorption ionization–time of flight mass spectrometry (MALDI-TOF MS). These two isolates were then used for genomic DNA extraction with the EZ1 BioRobot (Qiagen, Germany) using the EZ1 DNA tissue kit.

Antimicrobial susceptibility testing was performed on Mueller-Hinton agar by the disc diffusion method for all antimicrobials according to the EUCAST 2020 guidelines (https://www.eucast.org/fileadmin/src/media/PDFs/EUCAST_files/Breakpoint_tables/v_10.0_Breakpoint_Tables.pdf); Etest assays were additionally performed for ertapenem, imipenem, and fosfomycin. Among the tested antimicrobials, Marseille-Q1950 was susceptible only to fosfomycin, nitrofurantoin, and colistin. However, Marseille-Q1951showed resistance to fosfomycin (Table 1). Interestingly, intravenous fosfomycin therapy is not available in Lebanon.

**TABLE 1:** Antimicrobial susceptibility profiles of Marseille-Q1950 and Marseille-Q195 according to EUCAST 2020 guidelines

Isolate	Antimicrobial susceptibility profile^*a*^															
	AMX	AMC	PTZ	KF	CRO	FEP	ETP	IPM	CT	AK	CN	CIP	FF	F	DO	TSX
Marseille-Q1950	R	R	R	R	R	R	32 μg/ml^*b*^	4 μg/ml^*b*^	S	R	R	R	S	S	R	R
Marseille-Q1951	R	R	R	R	R	R	32 μg/ml^*b*^	4 μg/ml^*b*^	S	R	R	R	192 μg/ml^*b*^	S	R	S

a AMX, amoxicillin; AMC, amoxicillin-clavulanic acid; PTZ, piperacillin-tazobactam; KF, cephalothin; CRO, ceftriaxone; FEP, cefepime; ETP, ertapenem; IPM, imipenem; CT, colistin; AK, amikacin; CN, gentamicin; CIP, ciprofloxacin; FF, fosfomycin; F, nitrofurantoin; DO, doxycycline; TSX, trimethoprim-sulfamethoxazole; R, resistant; S, susceptible.

b MIC determined by Etest assay.

The genomic DNA of Marseille-Q1950 and Marseille-Q1951 was sequenced on the MiSeq platform (Illumina Inc., San Diego, CA, USA) by the paired-end strategy, as detailed previously (1, 2). The library was prepared following the workflow of the Nextera XT DNA library preparation kit (Illumina). Total information of 3.8 Gb was obtained with a cluster density of 416,000 clusters/mm^2^, with 91.7% of clusters passing quality control filters. Within this run, the index representations for strains Marseille-Q1950 and Marseille-Q1951 were 3.16% and 6.9%, respectively. The 7,995,112 paired-end reads were quality filtered using FastQC v0.11.8 (https://www.bioinformatics.babraham.ac.uk/projects/fastqc). We obtained 231,995 and 506,385 reads for strains Marseille-P1950 and Marseille-P1951, respectively. The forward and reverse strands were assembled using SPAdes v3.13 (3). Default parameters were used for all software. Marseille-Q1950 has a 5,181,515-bp-long genome assembled into 327 contigs (*N*_50_, 30,890 bp; *L*_50_, 50), with a G+C content of 50.44% and coverage of 13.36×. The genome of Marseille-Q1951 was assembled into 177 contigs (*N*_50_, 64,755 bp; *L*_50_, 22), with a size of 5,213,451 bp, coverage of 25.39×, and a G+C content of 50.46%. Genomic annotation was obtained using the NCBI Prokaryotic Genome Annotation Pipeline (PGAP) v5.1 (4). A total of 5,076 genes were identified, along with 3 rRNAs and 51 tRNAs.

Using a multilocus sequence typing (MLST) tool (https://github.com/tseemann/mlst), sequence type 405 (ST405) was assigned to Marseille-Q1950 and Marseille-Q1951. Using ResFinder v4.1 (5), we identified the drug resistance genes *aadA2*, *bla*_CTX-M-15_, *bla*_NDM-5_, *bla*_TEM-1B_, *catA1*, *dfrA12*, *rmtB*, *sul1*, and *tet*(B). Using PlasmidFinder v2.1 (6) with an identity threshold of 95%, we identified plasmids in both isolates, i.e., Col(BS512), IncFIA, IncFIB(AP001918), and IncFII(pAMA1167-NDM-5). Using CARD (7) with an identity threshold of 95%, we identified the fosfomycin resistance mutations *mdtG*, *glpT* (E448K), and *cyaA* (S352T) only in Marseille-Q1951.

### Data availability.

The draft genome and read sequences have been deposited in GenBank (BioProject number PRJNA697933) under the following accession numbers: JAFEVK000000000 and SRR13578895 for strain Marseille-Q1950 and JAFEVJ000000000 and SRR13578894 for strain Marseille-Q1951.
